# Chemotherapy for early-stage breast cancer: a brief history

**DOI:** 10.1038/sj.bjc.6605268

**Published:** 2009-09-15

**Authors:** M Verrill

**Affiliations:** 1 Northern Centre for Cancer Care, Freeman Hospital, High Heaton, Newcastle upon Tyne NE7 7DN, UK

**Keywords:** chemotherapy, adjuvant, anthracycline, docetaxel, neutropenia

## Abstract

The advent of chemotherapy for early-stage breast cancer has ushered in a new age of management for the condition. This article charts the evolution of chemotherapy for breast cancer, and highlights the current need for carefully planned, fully implemented local protocols to support the delivery of modern regimens.

Breast cancer has been recognised since at least 1600 BC, when an ancient Egyptian medical text (Smith, 2006) described eight cases of a tumour or ulcer of the breast that were treated by cauterisation. There followed many historical reports of the disease, all concluding that there was no cure.

In the seventeenth century, an understanding of the lymphatic circulation enabled the link to be made between the breast and the axillary lymph nodes, and led to the first lymph node surgery in women with breast cancer (Sakorafas, 2008). Radical surgery for breast cancer reached its zenith in the nineteenth century, at the hands of the US surgeon William Halsted, who removed not only the affected breast, but also the contralateral breast, all associated lymph nodes, and the underlying pectoral muscles (Halsted, 1907). This morbid and mutilating procedure was deemed necessary to prevent recurrence, but did little to change the natural history of the disease.

In the twenty-first century, breast cancer is the most common cancer in the United Kingdom, with in excess of 45 000 women and around 300 men diagnosed in 2005 (Cancer Research UK, 2009). However, the past half century has seen the emergence and evolution of new therapeutic approaches for breast cancer, including chemotherapy, radiotherapy and conservative surgery (Figure 1).

## The birth of chemotherapy

After the Second World War, the observation by Goodman and Gilman that nitrogen mustards had the potential for anticancer effects (Gilman, 1946), and parallel work on antifolates by Farber *et al* (1948), led to the first successful drug treatments for cancer (Goodman *et al*, 1984). Subsequently, observations of uracil uptake by normal rat mucosa and tumours led to the development of 5-fluorouracil (Heidelberger *et al*, 1957), and then cyclophosphamide+methotrexate+5-fluorouracil (CMF) – the first effective chemotherapy regimen for breast cancer (Bonadonna *et al*, 1976).

CMF was tested in the 1970s by Bonadonna *et al* (1976) and the Milan group. Their demonstration that the risk of breast cancer recurrence after surgery could be reduced with the addition of adjuvant chemotherapy paved the way for the development of surgical procedures less morbid than those pioneered by Halsted. Later, trials from the US National Surgical Adjuvant Breast and Bowel Project (NSABP) established that a combination of lumpectomy and radiotherapy was equivalent to mastectomy in terms of outcome (Fisher *et al*, 2002).

## New chemotherapy agents

Although the Milan group was the first to describe the use of an anthracycline (doxorubicin) in metastatic breast cancer (Bonadonna *et al*, 1969), the first anthracycline-containing regimen to become a ‘gold standard’ was doxorubicin+cyclophosphamide (AC), investigated initially by the NSABP in the 1990s (Fisher *et al*, 1990). The rationale for including the anthracycline was to reduce the duration of treatment, the number of hospital visits and the need for antiemetic medication (classical CMF involves 2 weeks of oral cyclophosphamide for each cycle, and produces significant and long-lasting nausea) (Fisher *et al*, 1990). There was no efficacy advantage of AC over CMF, possibly because of the shorter duration of treatment and the elimination of both 5-fluorouracil and methotrexate from the combination, and over the ensuing 30 years, CMF and AC became references for the development of newer, more effective chemotherapy regimens on both sides of the Atlantic.

In early attempts to improve the efficacy of CMF, a number of investigators tested regimens where, in a 6-cycle regimen, an anthracycline (doxorubicin or epirubicin) was substituted for methotrexate to make either FAC (5-FU+doxorubicin+cyclophosphamide) or FEC (5-FU+epirubicin+cyclophosphamide). Various doses of the drugs have been tested using either the classic 4-weekly schedule or a shortened 3-weekly regimen, which has often been compared with 3-weekly all intravenous CMF. The FAC regimen is most commonly 5-FU 500 mg m^–2^, doxorubicin 50 mg m^–2^ and cyclophosphamide 500 mg m^–2^, all 3 weekly (Martin *et al*, 2003). There are several FEC variations, leading to a degree of confusion when reference is made to the regimen, although there are several clearly defined schedules including ‘French FEC’ (French Adjuvant Study Group, 2001) and ‘Canadian FEC’ (Levine *et al*, 1998).

An alternative to the substitution of methotrexate is the addition of an anthracycline to CMF, in what is known as a block-sequential design. In the National Epirubicin Adjuvant Trial, four cycles of epirubicin were followed by four cycles of CMF, resulting in significantly improved efficacy compared with six cycles of CMF alone (Poole *et al*, 2006).

In the 1970s, the development of the taxanes, the first new cytotoxic drugs for several decades with activity in metastatic breast cancer (Wani *et al*, 1971), was soon followed by the inclusion of paclitaxel or docetaxel in various adjuvant chemotherapy trial regimens (Henderson *et al*, 2003; Mamounas *et al*, 2005; Martin *et al*, 2005; Bear *et al*, 2006). In the United States, AC followed by paclitaxel in a block-sequential design was shown to be more effective than AC alone (Henderson *et al*, 2003). This regimen was subsequently ‘accelerated’ – given every 2 weeks rather than every 3 weeks – an adaptation made possible through the use of granulocyte colony-stimulating factor, which helped to prevent chemotherapy-associated neutropenia (Citron *et al*, 2003). The accelerated approach resulted in a further increment in antitumour activity (Citron *et al*, 2003).

Another major development was marked by the Breast Cancer International Research Group (BCIRG)-001 trial, in which the 5-fluorouracil component of FAC was replaced by docetaxel, that is, the TAC regimen (Martin *et al*, 2005). The trial showed that TAC provided a significant improvement in efficacy compared with FAC. The French Adjuvant group modified FEC into a block-sequential regimen in which three cycles of FEC were followed by three cycles of docetaxel (FEC-T) (Roché *et al*, 2006). Since then, FEC-T has become a commonly used regimen following surgery for axillary lymph node-positive breast cancer in the United Kingdom.

## Current regimens for early-stage breast cancer

**Figure 2:**
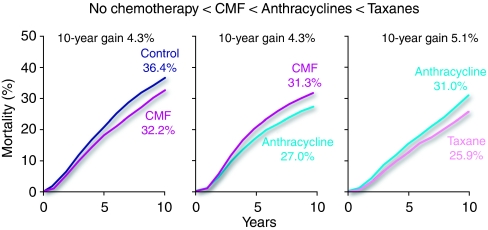
Stepwise improvements in efficacy of chemotherapy for early-stage breast cancer. Source: Preliminary data presented by R Peto at the 2007 San Antonio Breast Cancer Symposium on behalf of the Early Breast Cancer Trialists' Collaborative Group (EBCTCG).

There is currently no one gold standard regimen in early-stage breast cancer. The proliferation and diversity of trials, with varying interpretations of the standard of care, has led to endless conjecture on the best treatment, and much discussion of the route by which the ‘latest’ regimen to be tested has evolved. However, there is general agreement that CMF-like regimens are better than nothing, that anthracycline-containing regimens are better than CMF, and that the taxanes further add to the benefit of anthracyclines (Figure 2; Peto, 2007). The most robust evidence for this view comes from the Oxford overview of work by the Early Breast Cancer Trialists’ Collaborative Group, which includes the details of more than 250 000 women randomised into trials of polychemotherapy in early-stage breast cancer (Peto, 2007).

The latest guidelines on breast cancer management from the National Institute for Health and Clinical Excellence (NICE) emphasise the importance of chemotherapy for both early (NICE, 2009a) and advanced disease (NICE, 2009b). More specifically, for lymph node-positive early or locally advanced breast cancer, NICE states that docetaxel, not paclitaxel, should be part of the chemotherapy regimen (NICE, 2009a). NICE also recommends docetaxel monotherapy in patients with advanced breast cancer in whom anthracyclines have failed or are contraindicated, and in combination chemotherapy (e.g., with trastuzumab) in patients whose tumours overexpress human epidermal growth factor receptor 2 (HER2) (NICE, 2009b). NICE only recommends the combination of gemcitabine and paclitaxel as an option for advanced breast cancer if docetaxel monotherapy or the combination of docetaxel and capecitabine would also be appropriate. In the first instance, however, the guideline states that, in the majority of cases, patients should start treatment with taxane monotherapy (preferably docetaxel) followed by second-line vinorelbine or capecitabine monotherapy and then by third-line capecitabine or vinorelbine monotherapy (NICE, 2009b).

## Regimen delivery: practical and cost considerations

It is incumbent on the NHS to deliver treatments that are found to be effective in clinical trials, licensed by the European regulatory authorities and approved by NICE and the Scottish Medicines Consortium. Almost all NHS breast cancer units now have the facilities and expertise required to deliver complex chemotherapy regimens such as TAC. However, challenges remain, notably in service capacity, management and prevention of neutropenic complications, and the financial costs of chemotherapy.

Looking first at service capacity, it is clear that as thresholds for offering chemotherapy fall, the number of patients receiving treatment will increase. The introduction of trastuzumab as a routine adjuvant treatment for patients with breast cancers that overexpress HER2 has already stretched the system (NICE, 2009a), and there is concern that the growth in adjuvant therapies across cancer care as a whole will put increasing pressure on chemotherapy day units. Furthermore, there are now at least three studies, though the data are still preliminary, showing that the efficacy of standard breast cancer chemotherapy can be improved by the addition of a 6-monthly zoledronic acid infusion for 3 or 5 years (Coleman *et al*, 2006; Winter *et al*, 2008). Such an approach may save lives, but has the potential to more than double the number of intravenous treatments delivered to the population of patients with early-stage breast cancer, hence adding to the strain on resources.

The prevention and management of neutropenic events are covered in detail elsewhere in this supplement by Kelly and Wheatley, (2009); Cullen and Baijal, (2009) and Cameron, (2009). In summary, recent reports from the National Confidential Enquiry into Patient Outcome and Death (NCEPOD, 2008) and the National Chemotherapy Advisory Group (NCAG, 2009) have highlighted the need for clear, fully implemented local protocols for predicting, recognising and managing febrile neutropenia and neutropenic sepsis in patients receiving chemotherapy. In addition, there is international guidance on the use of G-CSF prophylaxis of neutropenia (Aapro *et al*, 2006; Smith *et al*, 2006).

Looking at the financial considerations, the drug acquisition cost for the UK-licensed TAC regimen is £6554 for six cycles, based on list prices, for a woman with a body surface area of 1.8 m^2^ (excluding VAT and assuming no drug wastage) (BNF, 2009). Six cycles of FAC for the same woman would cost £1278. Furthermore, in the BCIRG-001 trial, the febrile neutropenia rate for patients receiving TAC approached 25% (Martin *et al*, 2005), well above the recommended threshold for primary prophylaxis with G-CSF (Aapro *et al*, 2006; Smith *et al*, 2006). If the cost of a standard 6 mg dose of pegylated filgrastim is included, the drug acquisition cost increases to £10 839.

Some cancer networks have adopted FEC-T as a standard for patients with axillary lymph node-positive breast cancer, despite the lack of a specific licence for the regimen in the United Kingdom. The practice may be based on trial data suggesting that the rate of febrile neutropenia associated with FEC-T is lower than the 20% threshold for primary G-CSF prophylaxis stipulated by the international guidelines (Aapro *et al*, 2006; Roché *et al*, 2006; Smith *et al*, 2006), allowing services to avoid the cost of G-CSF prophylaxis. However, data from two separate UK studies, presented at the National Cancer Research Institute's Cancer Conference in 2008, do not support such an optimistic view of FEC-T's potential for reducing the risk of febrile neutropenia. Head *et al* (2008) reported a 25% rate of neutropenic fever in 137 patients receiving FEC-T; all affected patients were given secondary prophylactic G-CSF and there were no further episodes of sepsis. The authors, from hospitals across south-east England, recommend the use of primary prophylaxis in all patients receiving FEC-T. Ali *et al* (2008), from the Merseyside and Cheshire Cancer Network, reported a 27% rate of febrile neutropenia in 123 FEC-T recipients, including six patients who had two episodes . Only 8% of patients had further sepsis following secondary prophylaxis with G-CSF, and the authors suggest that primary or secondary G-CSF should be considered for all patients using FEC-T (Head *et al*, 2008).

## Conclusion

Although breast cancer remains a common malignancy, the outlook for women with early-stage disease has been transformed since the Halsted days when radical surgery was the only therapeutic option. Recent advances in chemotherapy for breast cancer have culminated, in England and Wales, in the latest NICE guidelines for the management of early and advanced disease. However, effective use of UK-licensed and NICE-approved regimens requires the development and full implementation of local policies aimed not only at treatment delivery but also at strategies for predicting, preventing and managing the complications of chemotherapy, notably febrile neutropenia. Such policies, in operation across every cancer network, would save lives and, I believe, reinforce clinicians’ confidence in the regimens now at our disposal.

## Figures and Tables

**Figure 1:**
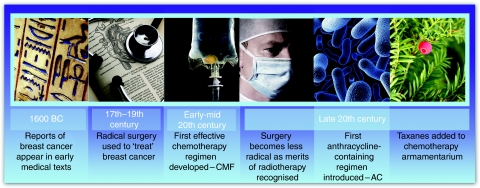
Chemotherapy evolution timeline.
